# Carcinoma cells that have undergone an epithelial-mesenchymal transition differentiate into endothelial cells and contribute to tumor growth

**DOI:** 10.18632/oncotarget.27940

**Published:** 2021-04-13

**Authors:** Nathalie Sphyris, Cody King, Jonathan Hoar, Steven J. Werden, Geraldine V. Vijay, Naoyuki Miura, Akhilesh Gaharwar, Tapasree Roy Sarkar

**Affiliations:** ^1^Department of Translational Molecular Pathology, The University of Texas MD Anderson Cancer Center, Houston, TX, USA; ^2^Department of Biochemistry, Texas A&M University, College Station, TX, USA; ^3^Department of Biology, Texas A&M University, College Station, TX, USA; ^4^Department of Biochemistry, Hamamatsu University School of Medicine, Hamamatsu, Japan; ^5^Department of Biomedical Engineering, Texas A&M University, College Station, TX, USA; ^6^Present address: Cancer Research UK Beatson Institute, Glasgow G61 1BD, UK; ^*^These authors contributed equally to this work

**Keywords:** angiogenesis, endothelial transdifferentiation, epithelial-mesenchymal transition, vasculogenic mimicry, FOXC2

## Abstract

Hypoxia stimulates neoangiogenesis, promoting tumor outgrowth, and triggers the epithelial-mesenchymal transition (EMT), which bestows cells with mesenchymal traits and multi-lineage differentiation potential. Here, we investigated whether EMT can confer endothelial attributes upon carcinoma cells, augmenting tumor growth and vascularization. Following orthotopic implantation of MCF-7 human epithelial breast cancer cells into mice, tumors of different sizes were immunostained for markers of hypoxia and EMT. Larger tumors were well-vascularized with CD31-positive cells of human origin. Hypoxic regions, demarcated by HIF-1α staining, exhibited focal areas of E-cadherin loss and elevated levels of vimentin and the EMT-mediator FOXC2. Implantation of MCF-7 cells, co-mixed with human mammary epithelial (HMLE) cells overexpressing the EMT-inducer Snail, markedly potentiated tumor growth and vascularization, compared with MCF-7 cells injected alone or co-mixed with HMLE-vector cells. Intra-tumoral vessels contained CD31-positive cells derived from either donor cell type. FOXC2 knockdown abrogated the potentiating effects of HMLE-Snail cells on MCF-7 tumor growth and vascularization, and compromised endothelial transdifferentiation of mesenchymal cells cultured in endothelial growth medium. Hence, cells that have undergone EMT can promote tumor growth and neovascularization either indirectly, by promoting endothelial transdifferentiation of carcinoma cells, or directly, by acquiring an endothelial phenotype, with FOXC2 playing key roles in these processes.

## INTRODUCTION

Angiogenesis is a normal physiological process that entails the development of new blood vessels through remodeling of a pre-existing vasculature, underpinned by endothelial cell sprouting, proliferation, and fusion [1, 2]. The activation of normally quiescent endothelial cells, following exposure to angiogenic stimuli, lays the foundation for the formation of the vascular network during embryonic development and facilitates wound-healing post injury. Notably, the ability of solid tumors to induce and sustain angiogenesis—often termed neoangiogenesis in the cancer context—has been recognized as one of the distinguishing hallmarks of cancer [3]. Accordingly, the induction of angiogenesis enables tumor growth beyond a certain critical size (1–2 mm^3^), when the pre-existing tissue vasculature becomes inadequate to support the ever-increasing growth demands of the tumor [4, 5]. In addition to ensuring the availability of essential nutrients and oxygen, the establishment of new blood vessels facilitates the removal of cytotoxic metabolic by-products and carbon dioxide and, crucially, enables shed tumor cells to disseminate to distant sites [3].

At the molecular level, an important nexus in tumor progression is the activation of the “angiogenic switch,” governed by the balance between a multitude of pro- and anti-angiogenic factors, which regulate endothelial cell proliferation and migration [2, 6]. The dominance of pro-angiogenic factors promotes the growth of new blood vessels from pre-existing vessels through so-called sprouting angiogenesis [2, 6]. In addition, bone marrow-derived endothelial progenitor cells can be mobilized to initiate *de novo* vessel formation in response to angiogenic signals (vasculogenesis) [7]. Together, these angiogenic mechanisms facilitate the progression from avascular hyperplasia to a highly vascularized outgrowing tumor [2, 6]. However, many avascular tumors can nevertheless grow, at least initially, without evoking an angiogenic response through a process known as vessel co-option. In this non-angiogenic mode, which mostly prevails in highly vascularized host tissues, tumor cells hijack the host vasculature and migrate along pre-existing blood vessels, thus invading the surrounding tissue [8, 9]. A fourth mechanism—termed vasculogenic mimicry—entails the *de novo* generation of microvessels, lined with highly invasive tumor cells embedded in a rich extracellular matrix, essentially mimicking a true vascular endothelium and, notably, lacking in the endothelial cell markers CD31 and CD34 [10, 11]. Finally, newly formed blood vessels may emerge through transdifferentiation of neoplastic or tumor stem-like cells into CD31-positive endothelial-like cells, as has been documented in neuroblastoma, B-cell lymphoma, and glioblastoma [12–16]. Therefore, the complex mechanisms underlying neoangiogenesis differ from physiological angiogenesis and lead to the formation of dysfunctional and disorganized vessels with a defective endothelial layer, which nevertheless fuels tumor progression.

As rapidly developing tumors outgrow their blood supply, they begin to exhibit areas of localized oxygen deprivation, a condition known as hypoxia [17]. Hypoxia, in turn, tilts the balance towards pro-angiogenic factors, activating the angiogenic switch and perpetuating a vicious cycle of hypoxia-neoangiogenesis [17, 18]. In addition to triggering angiogenesis, hypoxia is one of the most potent inducers of the epithelial-mesenchymal transition (EMT) [19–21], a latent embryonic program that is misappropriated during carcinoma progression. EMT is a complex series of cellular-reprogramming events that facilitate the conversion of immotile, polarized epithelial cells into intrinsically migratory and invasive mesenchymal counterparts [22–24]. Recent studies have further established that EMT bestows stem-like properties upon differentiated epithelial carcinoma cells, conferring a stem cell-associated CD44^high^/CD24^low^ antigenic phenotype, tumor-initiating capabilities, and intrinsic resistance to chemotherapy [25, 26].

We and others previously identified the transcription factor Forkhead Box C2 (FOXC2) as a key downstream effector of several converging EMT pathways that functions to confer the mesenchymal and stem cell traits underpinning metastatic competence [27–29]. FOXC2 has also been linked to angiogenesis—both in the context of normal development and tumor progression—through its ability to transcriptionally regulate genes encoding pro-angiogenic factors including OPN [30], VEGF-A [31], ITGB-3 [32, 33], HEY-2 [33], PDGF-β [34], DLL-4 [32, 33], CXCR-4 [32], and ANG-2 [35]. In addition, subcutaneous injection of B16 melanoma cells into *Foxc2* haploinsufficient mice has been shown to lead to the impaired formation of tumor blood vessels and, accordingly, compromise tumor growth [34, 36]. Thus, FOXC2 has emerged as an important transcriptional regulator of tumor cell-intrinsic and -extrinsic factors influencing EMT and angiogenic remodeling during tumor progression.

We previously reported that epithelial cells induced to undergo EMT exhibit multi-lineage differentiation potential, similar to mesenchymal stem cells, thus displaying the ability to differentiate into three major mesodermal lineages: osteoblasts, adipocytes, and chondrocytes [37]. Given the inherent plasticity of cells that have undergone EMT and the involvement of hypoxia in EMT and angiogenesis, we sought to ascertain whether cells, undergoing EMT in the hypoxic milieu, can acquire endothelial cell attributes and augment tumor growth by directly contributing to the tumor vasculature. Our findings link the stemness, conferred through EMT, to the acquisition of endothelial cell traits and the augmentation of tumor angiogenesis in a FOXC2-dependent manner.

## RESULTS

### Hypoxia promotes EMT, endothelial transdifferentiation, and vascularization in outgrowing MCF-7 tumors

**Figure 1:**
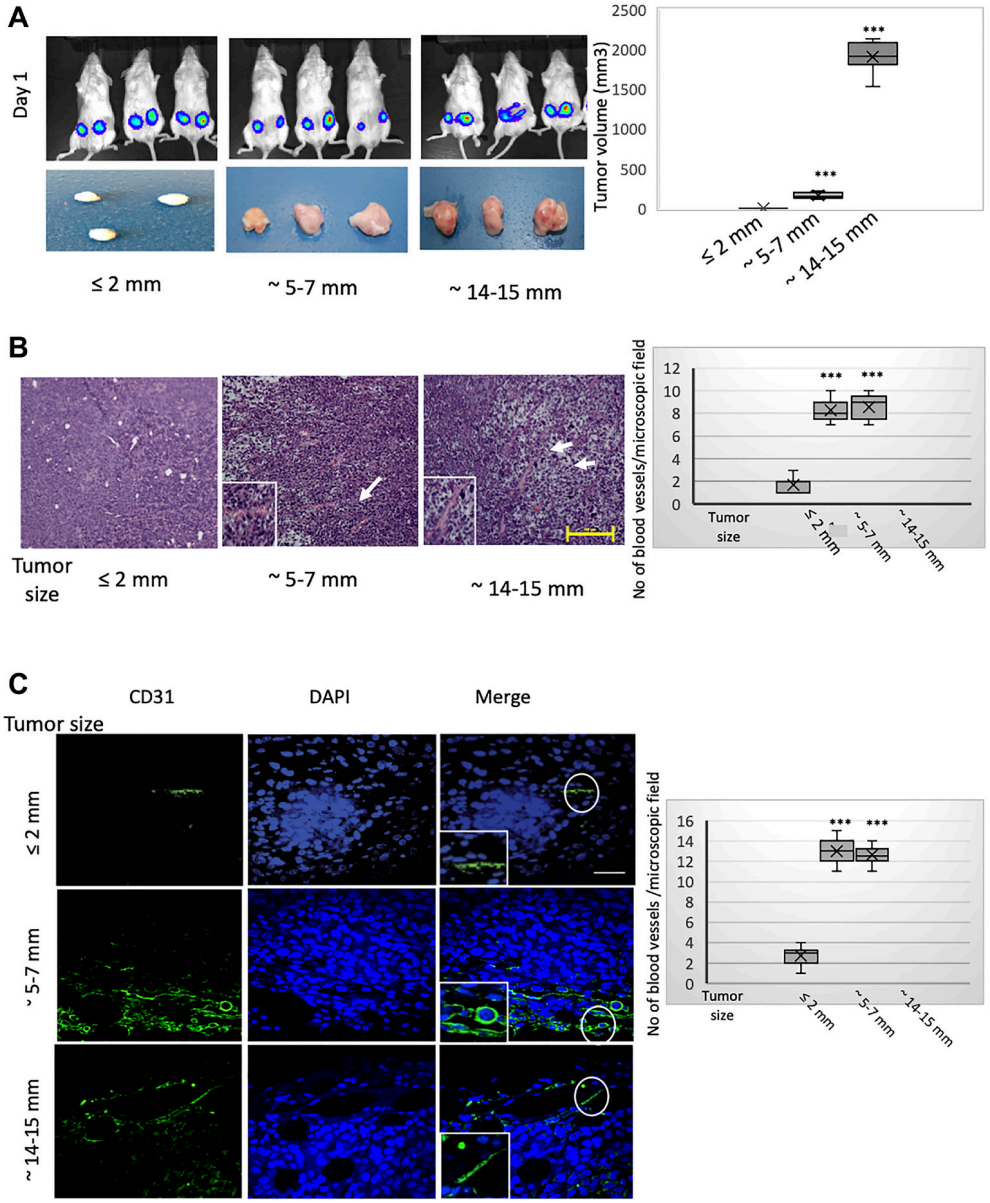
MCF-7 tumors of different sizes exhibit progressively developed vasculatures incorporating CD31-positive cells of human donor origin. RFP/luciferase-labeled MCF-7 cells were orthotopically implanted into recipient NOD/SCID hosts. Tumors were allowed to grow to different sizes over time and harvested when their size reached approximately ≤ 2 mm, 5–7 mm, or 14–15 mm in longitudinal diameter (corresponding to 2, 6, and 10 weeks post implantation, respectively). Tumor growth was monitored weekly using caliper measurements and bioluminescent imaging. As MCF-7 cells are only weakly tumorigenic, some of the implanted tumors had developed only sub-millimeter primary tumors at necropsy that were too small to be excised and analyzed. *n* = 5 mice per group (2 implantations into contralateral glands per mouse). (**A**) Contralateral orthotopic injections of 1 × 10^6^ MCF-7 cells yielded bioluminescent signals of similar intensity as imaged on day 1 post implantation (top panels). Macroscopic images of the resulting tumors of different sizes, measuring approximately ≤ 2 mm, 5–7 mm, or 14–15 mm in longitudinal diameter, and harvested 2, 6, and 10 weeks post implantation, respectively (bottom panels). The estimated volumes of the tumors, grouped according to longitudinal diameter, are represented graphically (right). The box and whisker plots show the median tumor volume per group (horizontal bar within the box), 25% percentile (bottom of box), 25th to the 75th percentile (top of box), and whiskers which extend an additional 1.5× the interquartile range. ^***^ *P* < 0.001; Student’s *t-test*, two-tailed. (**B**) Representative hematoxylin and eosin (H&E) staining of sections from the core regions of the primary tumors described in (A). The location of blood vessels is indicated by arrows. Scale bar, 100 μm. The average number of blood vessels per microscopic field of the H&E-stained tumor sections is plotted on the right. (**C**) Representative immunofluorescent staining of the core regions of the tumors described in (A), using an antibody recognizing human CD31 (green). Nuclei were counterstained with DAPI (blue). Right panels are merged images of individual channels. The boxed areas represent high-magnification images of encircled areas. Scale bar, 100 μm. Representative images are shown. The average number of CD31-positive structures per microscopic field according to tumor size (longitudinal diameter) is plotted on the right. Student’s *t-test* was performed to test significance. Data indicate mean ± SEM. *n* = 5. ^*^ *P* < 0.05, ^**^ *P* < 0.01, ^***^ *P* < 0.001.

Using MCF-7 cells, a weakly tumorigenic human breast epithelial adenocarcinoma cell model, we first determined the relationship between tumor size, the establishment of a tumor vasculature, and the induction of hypoxia and EMT within the tumor core. For this, we orthotopically implanted 1 × 10^6^ RFP/luciferase-labeled MCF-7 cells into the contralateral pair of fourth mammary fat-pads of female NOD/SCID mice (Figure 1A, top panels) and monitored tumor progression weekly using caliper measurements and bioluminescence. At appropriate time-intervals post implantation, allowing for the formation of palpable tumors measuring approximately ≤ 2 mm, 5–7 mm, or 14–15 mm in longitudinal diameter (2, 6, and 10 weeks post implantation, respectively), we sacrificed five mice per timepoint and removed the tumors (Figure 1A, bottom panels). We recorded the longitudinal and transverse diameters of the excised tumors and used the measurements to estimate the corresponding tumor volumes (Figure 1A, right). As MCF-7 cells are only weakly tumorigenic, some of the tumors (sub-millimeter) were too small to be analyzed at necropsy. We observed that the tumors measuring ≤ 2 mm in longitudinal diameter were relatively pale in appearance, indicative of a markedly reduced vascularity, compared with the pink/reddish color of tumors measuring 5–7 mm and 14–15 mm (Figure 1A, bottom panels). The hypovascularity of the ≤ 2 mm-sized tumors was evident in the corresponding hematoxylin and eosin-(H&E)-stained tissue sections (Figure 1B), especially when compared to tumors measuring 5–7 mm or 14–15 mm, which exhibited progressively developed vascular networks (Figure 1B). Indeed, the location of the blood vessels in the latter two sets of tumors (indicated by arrows in Figure 1B) is adjacent to necrotic regions, consistent with the stimulation of vascularization to alleviate the hypoxic conditions within the tumor core. To distinguish vessels of human tumor cell origin from host-derived counterparts, we performed immunofluorescence with a human-specific CD31 antibody (Figure 1C). Whereas only occasional CD31-positive cells were observed in tumors measuring ≤ 2 mm, CD31 immunostaining strongly decorated numerous blood vessels within the core regions of tumors sized 5–7 mm or 14–15 mm (Figure 1C). On the basis of the contiguous staining patterns, and given that the anti-CD31 antibody employed is human-specific, we concluded that neoangiogenesis in the core regions of these outgrowing tumors is enacted predominantly through MCF-7 cell transdifferentiation towards an endothelial phenotype.

**Figure 2:**
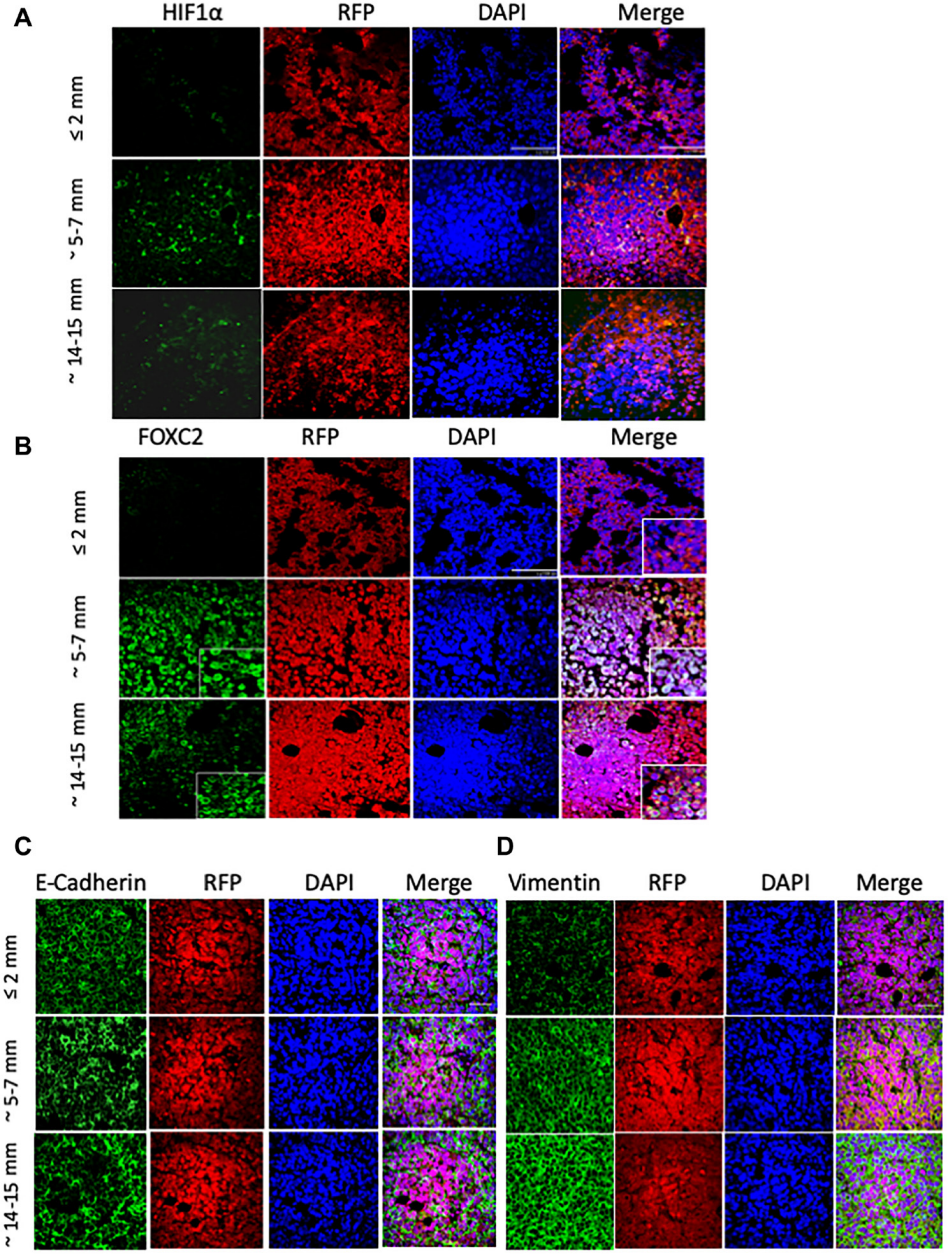
MCF-7 tumor outgrowth is accompanied by increased expression of markers of EMT and hypoxia. RFP/luciferase-labeled MCF-7 cells were orthotopically injected into recipient NOD/SCID hosts, and tumors were allowed to grow to until their size reached approximately ≤ 2 mm, 5–7 mm, or 14–15 mm in longitudinal diameter. *n* = 5 mice per group. The excised tumors (shown in Figure 1A) were fixed in formalin, paraffin-embedded, and sectioned (5 microns thick) prior to immunostaining with the indicated primary antibodies against: HIF-1α (**A**), FOXC2 (**B**), E-cadherin (**C**), and vimentin (**D**), each pseudo-colored green in the respective images. Dual staining with anti-RFP antibody (red) confirms that the stained tumor cells originate from the injected MCF-7 cells and not the mouse hosts. Nuclei were counterstained with DAPI (blue). Right panels are merged images of individual channels. Note the encircled areas displaying localized loss of E-cadherin immunoreactivity in tumors sized 5–7 mm and 14–15 mm. Scale bars, 100 μm. Representative images are shown.

It is well established that hypoxia comprises a potent stimulus for tumor angiogenesis [17]. One of the key regulators of the hypoxic response is hypoxia-inducible factor 1 (HIF-1), a pleiotropic transcription factor that promotes tumor cell proliferation, survival, migration, and angiogenesis in response to low oxygen tension [38–40]. The expression of the gene encoding the HIF-1α subunit is induced under hypoxia, whereupon the newly-translated HIF-1α protein associates with the constitutively expressed HIF-1β subunit, forming the active HIF-1 heterodimer. Accordingly, we detected intense HIF-1α staining within the cores of the tumors sized 5–7 mm (Figure 2A, middle panels), presumably demarcating hypoxic regions. Intriguingly, HIF-1α immunoreactivity was predominantly localized to the cytoplasm of tumor cells, and exhibited a punctate staining pattern with occasional speckles noted in nuclei. Consistent with this staining pattern, it was recently proposed that HIF-1α may translocate to the mitochondria and directly alter mitochondrial metabolism, in a manner independent of its activity as a transcription factor [41]. Accordingly, HIF-1α was localized to the mitochondria of cultured cardiac myocytes, subjected to hypoxia preconditioning consisting of 4 cycles of 1-hour hypoxia and 1-hour re-oxygenation [41–43], which we would argue better recapitulates the alternating cycles of hypoxia and re-oxygenation seen in human tumors. A cytoplasmic staining pattern for HIF-1α has also been documented in other tumor types, where it seems to be associated with more differentiated epithelial carcinoma cells [43–45]. However, the functional significance of this expression pattern *in vivo* remains poorly understood.

The heterogeneous, diffuse, and somewhat diminished HIF-1α staining pattern in the 14–15 mm-sized tumors (Figure 2A, bottom panels), compared to tumors sized 5–7 mm, conceivably indicates that the oxidative stress may have diminished due to the establishment of a vascular network ameliorating the oxygen deficit. On the other end of the spectrum, tumors measuring ≤ 2 mm in diameter exhibited HIF-1α immunoreactivity only in occasional cells, consistent with HIF-1α stabilization in areas of localized oxygen deprivation (Figure 2A, top panels).

Given the involvement of hypoxia and HIF-1α in the induction of EMT [20, 46], we next examined the expression of FOXC2 (Figure 2B), a transcription factor that functions as a common denominator of multiple EMT programs [28]. Whereas FOXC2 immunostaining was noticeably absent from MCF-7 tumors measuring ≤ 2 mm (Figure 2B, top panels), its expression was significantly up-regulated across the cores of tumors sized 5–7 mm and, to a lesser degree, 14–15 mm (Figure 2B, middle and bottom panels, respectively). Indeed, a significant proportion of cells, within the 5–7 mm tumor cores, displayed nuclear localization of FOXC2, consistent with its activation as a transcription factor and the engagement of the EMT program in response to hypoxic conditions in the outgrowing tumors.

We further used immunofluorescence to examine the expression of E-cadherin in this series of MCF-7 tumors allowed to grow to different sizes. Irrespective of tumor size, the compacted tumor cores exhibited a characteristic honeycomb-like staining pattern for E-cadherin, consistent with its localization predominantly at the cell membrane (Figure 2C). Interestingly, however, the core regions of the tumors, measuring 5–7 mm and 14–15 mm, displayed localized loss of E-cadherin immunoreactivity, suggesting that these areas may represent the initiation of EMT events leading to regional loss of cell-cell cohesion and local invasion (Figure 2C). Conversely, the staining intensity of the mesenchymal marker vimentin progressively and markedly increased within the compacted cellular cores of the tumors sized 5–7 mm and 14–15 mm (Figure 2D, middle and bottom panels). In stark contrast, tumors measuring ≤ 2 mm, exhibited low baseline vimentin expression (Figure 2D, top panels). Interestingly, although the intensity of vimentin staining varied somewhat in tumors sized 5–7 mm and 14–15 mm, its expression was increased in all constituent cells unlike the loss of E-cadherin from discrete foci. Overall, the above findings suggest that as MCF-7 tumors outgrow their blood supply, they develop hypoxic core regions, demarcated by HIF-1α accumulation, wherein cells exhibit phenotypic changes consistent with the initiation of EMT as well as transdifferentiation to a CD31-positive endothelial cell lineage.

We next examined the infiltration of the differently sized tumors by myeloid-derived suppressor cells (MDSCs), a heterogeneous population of bone marrow-derived myeloid progenitors known to be recruited to hypoxic tumor areas [47]. Overexpression of HIF-1α causes tumor cells to release chemoattractants and chemokines that can recruit MDSCs to hypoxic regions [47–49]. MDSCs can, in turn, suppress the anti-tumor immune response and secrete pro-angiogenic factors, supporting angiogenesis, tumor invasiveness, and tumor progression [50]. In addition, CD11b-Gr-1+ MDSCs can directly incorporate into the tumor endothelium and express CD31 [48]. Using immunofluorescence, we found that tumors sized 5–7 mm were infiltrated by more Gr1+ cells compared with tumors of the other sizes (Supplementary Figure 1), which is consistent with higher levels of hypoxia and neovascularization in these intermediate-sized tumors.

### Cells that have undergone EMT promote neovascularization and tumor outgrowth of admixed MCF-7 cells

Given that hypoxia has been linked to the induction of both EMT and angiogenesis [17], we reasoned that the emergence of cells that have undergone EMT, within the hypoxic tumor microenvironment, may potentiate the outgrowth and vascularization of MCF-7 tumors. To model such an eventuality, we first tested whether the co-injection of cells, experimentally induced to undergo EMT *in vitro*, impacted the ability of MCF-7 cells to form tumors in NOD/SCID mice. For this, we employed a well-established model of human mammary epithelial cells (HMLE) immortalized using the genes encoding hTERT, the catalytic subunit of human telomerase, and the SV40 large-T antigen, an inhibitor of p53-dependent transactivation [51]. To model mesenchymal cells generated through EMT, we used HMLE cells enforced to undergo EMT via ectopic expression of the EMT-inducing transcription factors Snail (HMLE-Snail) or Twist (HMLE-Twist), with vector-transduced counterparts (HMLE-vector) serving as epithelial controls. Importantly, HMLE cells are not inherently tumorigenic as they are immortalized but not transformed [51]. Moreover, while ectopic expression of Snail or Twist in HMLE cells elicits EMT, HMLE-Snail and HMLE-Twist cells fail to initiate tumors efficiently *in vivo*, since tumorigenic potential in this context is bestowed only upon ectopic expression of *RAS* or other oncogenic drivers [25, 51].

**Figure 3:**
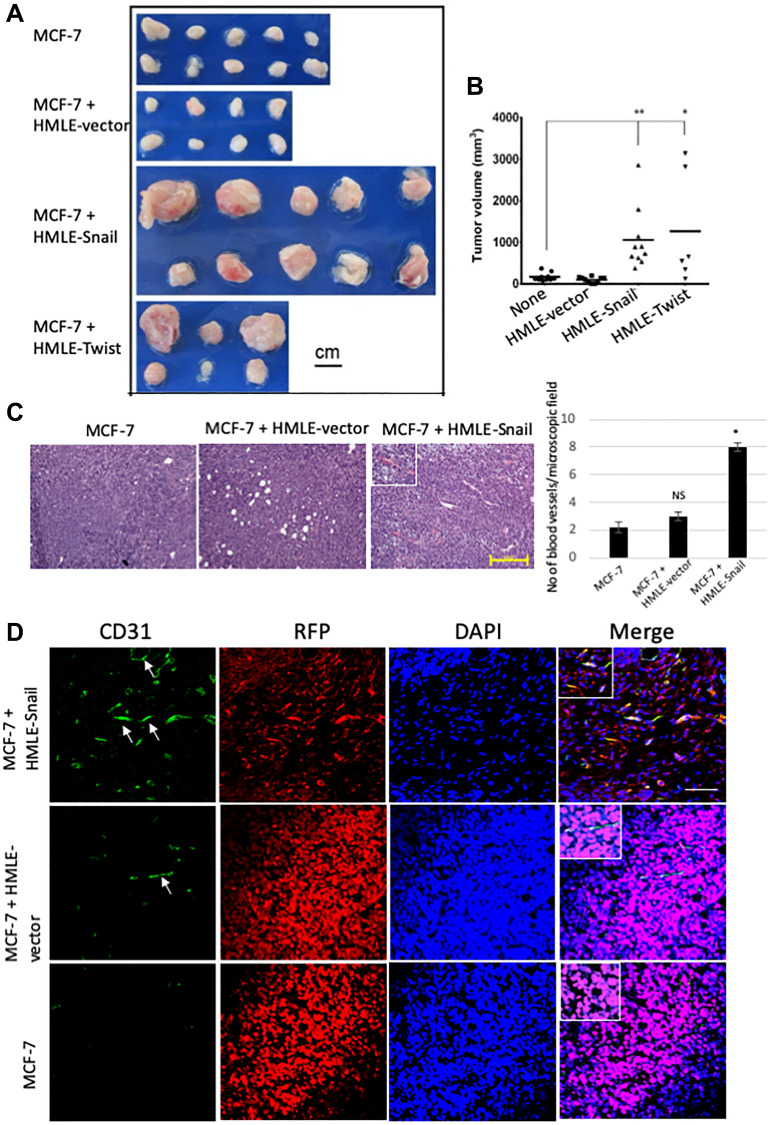
Cells that have undergone EMT promote endothelial transdifferentiation, neovascularization, and outgrowth of admixed MCF-7 cells. RFP/luciferase-labeled MCF-7 cells (0.5 × 10^6^) were co-mixed with MCF-7, HMLE-vector, HMLE-Snail, or HMLE-Twist cells at a 1:1 ratio (0.5 × 10^6^ cells per cell type) and orthotopically implanted into female NOD/SCID mice. The mice were sacrificed 5 weeks post implantation, and the primary tumors were excised. *n* = 5 mice per group. (**A**) Macroscopic images of the primary tumors from mice injected with admixtures of 0.5 × 10^6^ MCF-7 cells with 0.5 × 10^6^ MCF-7, HMLE-vector, HMLE-Snail, or HMLE-Twist cells. In the experiment presented, one mouse injected with MCF-7/HMLE-vector cells developed sub-millimeter tumors that were too small to be analyzed at necropsy, and two mice injected with MCF-7/HMLE-Twist cells succumbed to their tumor burden before the end of the 5-week study duration. Note the pink/reddish color of MCF-7/HMLE-Snail and MCF-7/HMLE-Twist tumors compared with the pale appearance of tumors formed by MCF-7 cells injected either alone or admixed with HMLE-vector cells. Scale bar, 1 cm. (**B**) The size of the excised primary tumors, shown in (A), was measured with a caliper, and the tumor volume (mm^3^) was calculated using a modified ellipsoid formula as described in Materials and Methods. The calculated tumor volumes (y-axis) are plotted against the composition of the implanted admixtures (x-axis) of MCF cells with: MCF-7 cells alone (none), HMLE-vector (HMLE), HMLE-Snail (HMLE-Snail), or HMLE-Twist (HMLE-Twist). Horizontal bars indicate the mean tumor volume. (**C**) Representative H&E staining of sections from the core regions of the primary tumors, harvested 5 weeks post implantation as described in (A). The location of blood vessels is indicated by arrows. Scale bar, 100 μm. The average number of blood vessels per microscopic field is shown on the right. (**D**) Representative immunofluorescent staining of the core regions of the primary tumors, harvested 5 weeks post implantation as described in (A), using antibodies recognizing human CD31 (green) and RFP (red). Nuclei were counterstained with DAPI (blue). Right panels are merged images of individual channels. Insets represent images of selected regions. Scale bar, 100 μm. Representative images are shown. Student’s *t-test* was performed to test significance. Data indicate mean ± SEM. *n* = 5. ^*^ *P* < 0.05, ^**^ *P* < 0.01, ^***^ *P* < 0.001.

In order to assess the contribution of cells that have undergone EMT to tumor growth, we co-mixed RFP/luciferase-labeled MCF-7 cells with HMLE-vector, HMLE-Snail, or HMLE-Twist cells, at a 1:1 ratio (0.5 × 10^6^ cells per cell type), and orthotopically implanted these admixtures into female NOD/SCID mice. MCF-7 cells, admixed with HMLE-Snail or HMLE-Twist cells, generated tumors of significantly greater volume, compared with a homogeneous MCF-7 cell suspension (1 × 10^6^ cells) or MCF-7 cells co-mixed 1:1 with epithelial HMLE-vector cells (Figure 3A and 3B). Additionally, injection of MCF-7 cells, co-mixed with either HMLE-Snail or HMLE-Twist cells, generated tumors with a markedly increased vasculature, as indicated by their pink/reddish color. This is in stark contrast with the pale appearance of tumors formed by MCF-7 cells either injected alone or admixed with epithelial HMLE-vector cells (Figure 3A). To follow up on this observation, we next assessed the formation of a tumor-associated vasculature by examining H&E sections from the harvested tumor cores. Histological analysis revealed extensive vascularization in tumors formed by co-injection of HMLE-Snail and MCF-7 cells (Figure 3C). Confirming our earlier observations that MCF-7 cells can transdifferentiate into endothelial cells *in vivo*, we detected numerous CD31-positive cells that also stained positive for RFP in the admixed MCF-7/HMLE-Snail tumors (Figure 3D). These RFP/CD31 double-positive cells appeared elongated and flattened, with a morphology similar to *bona fide* endothelial cells (Figure 3D, indicated by arrows), and were observed to form luminal structures, when viewed in cross-section (Figure 3D, indicated by an arrow). Importantly, RFP/CD31 double-positive cells were most prominent in the large tumors, formed by admixed MCF-7 and HMLE-Snail cells (Figure 3D), suggesting that HMLE-Snail cells in the tumor milieu can stimulate MCF-7 cell growth and endothelial transdifferentiation, despite their own inherent lack of tumorigenic potential. Conversely, the significantly diminished numbers of CD31-positive cells, observed in the small tumors formed by MCF-7 cells injected either alone or admixed with HMLE-vector cells, reinforce the assertion that the capacity to potentiate endothelial transdifferentiation is predominantly associated with the mesenchymal phenotype of the cell input in this model.

**Figure 4:**
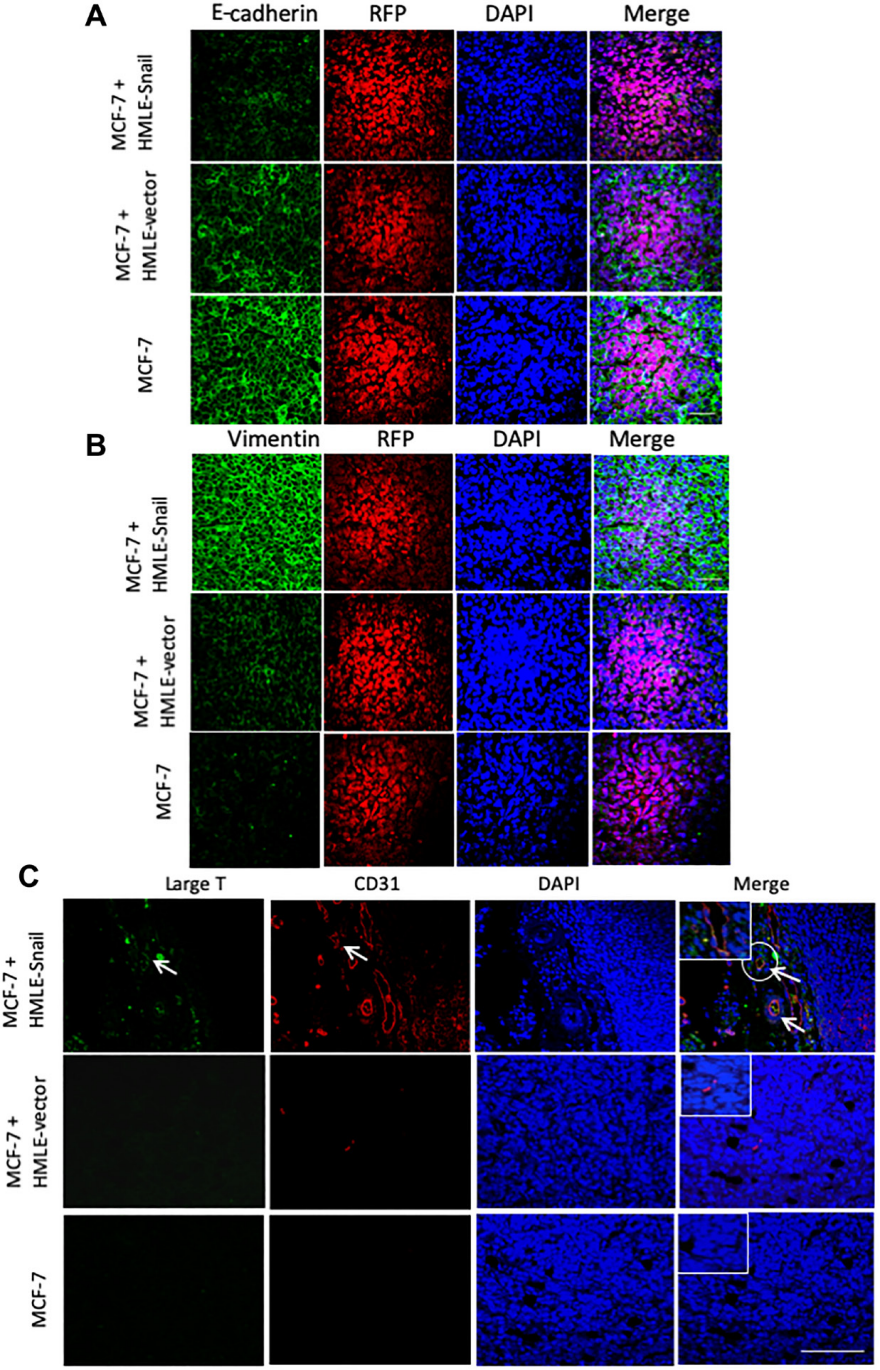
HMLE-Snail cells gain CD31 expression and promote acquisition of mesenchymal traits by admixed MCF-7 cells. RFP/luciferase-labeled MCF-7 cells (0.5 × 10^6^) were co-mixed with 0.5 × 10^6^ MCF-7, HMLE-vector, or HMLE-Snail cells, and orthotopically implanted into female NOD/SCID mice. Ten weeks post implantation, the tumors were harvested and processed for immunofluorescent staining. *n* = 5 mice per group. (**A**, **B**) Tumor core sections were co-stained with antibodies directed against RFP (red) and either E-cadherin (A) or vimentin (B), both pseudo-colored green. Nuclei were counterstained with DAPI (blue). Right panels are merged images of individual channels. Note the progressive reduction of E-cadherin expression, commensurate with the attenuation of the honeycomb-like membrane staining pattern across these tumor cores and the inversely-correlated augmented vimentin staining. (**C**) Sections from the tumor cores were co-stained with antibodies directed against the SV40 large-T antigen (green) and human CD31 (red). Nuclei were counterstained with DAPI (blue). Right panels are merged images of individual channels. Arrows indicate SV40 large-T antigen/CD31 double-positive cells lining the lumens of vascular structures in tumors formed by admixed MCF-7/HMLE-Snail cells. Boxed areas represent high-magnification images of selected encircled areas. Scale bars, 100 μm. Representative images are shown.

Since our data from the MCF-7 tumors, allowed to grow to different sizes, raise the possibility that epithelial cells can undergo EMT and subsequently differentiate into CD31-positive cells, we next investigated the staining patterns of E-cadherin and vimentin, markers of the epithelial and mesenchymal phenotypes, respectively. We found that RFP-positive MCF-7-derived cells, existing within MCF-7/HMLE-Snail tumors, exhibited markedly diminished levels of membrane-localized E-cadherin, and significantly increased expression of vimentin, consistent with the induction of EMT (Figure 4A and 4B, top panels). On the other hand, RFP-positive cells in MCF-7/HMLE-vector tumors largely retained the honeycomb-like staining pattern for E-cadherin, albeit with a moderately reduced staining intensity, and displayed only a slight upregulation of vimentin, compared with tumors formed by MCF-7 cells injected alone (Figure 4A and 4B, middle and bottom panels). These staining patterns are consistent with the progressive induction of a partial EMT in the MCF-7 cell compartment of this series of tumors, which is dramatically augmented by the presence of cells that have already undergone EMT and/or initiated as tumors increase in size.

As mentioned above, CD31 immunostaining decorated numerous blood vessels in the tumors formed by admixed MCF-7 and HMLE-Snail cells (Figures 3D and 4C). Since cells that have undergone EMT exhibit intrinsic cellular plasticity [25, 26] and the capacity to transdifferentiate into mesodermal lineages [37], we next investigated whether HMLE-Snail cells could directly contribute to the tumor vasculature in this model. For this, we co-stained tumor sections with antibodies directed against the SV40 large-T antigen, used to immortalize the HMLE cell-line series, and human CD31, to mark endothelial-like cells. Strikingly, we detected SV40 large-T/CD31 double-positive cells in tumors, formed by admixed MCF-7/HMLE-Snail cells, which had been incorporated into vascular endothelial structures forming distinct lumens (Figure 4C, indicated by arrows). This suggests that cells that have undergone Snail-induced EMT can acquire CD31 expression *in vivo* and can directly contribute to the lining of intra-tumoral blood vessels. This finding is consistent with the well-established stem-like capabilities of cells that have passed through an EMT and extends their differentiation potential to include the endothelial lineage.

We also detected rare, elongated, CD31-positive/RFP-negative cells in MCF-7/HMLE-vector tumors, which were presumably derived from HMLE-vector cells (data not shown). Conversely, in MCF-7/HMLE-vector tumor sections stained for SV40 large-T antigen, we detected rare CD31-positive cells that did not co-express SV40 large-T antigen, suggesting that they originated from MCF-7 cells (data not shown). Together, our findings suggest that the ability to potentiate tumor growth and augment tumor vascularization is predominantly associated with the highly-plastic mesenchymal HMLE-Snail cells. However, we postulate that MCF-7 or HMLE-vector cells could generate endothelial progeny under the proviso that they are first induced to undergo EMT within the hypoxic microenvironment of the outgrowing tumor. Overall, our results suggest that cells that have undergone EMT may potentiate neoangiogenesis either indirectly, by influencing the tumor microenvironment to elicit the dedifferentiation and transdifferentiation of epithelial tumor cells towards an endothelial-like phenotype, or directly, by undergoing endothelial differentiation and integrating into the nascent tumor vasculature.

### Cells that have undergone EMT can acquire endothelial traits and functional behaviors *in vitro*

**Figure 5:**
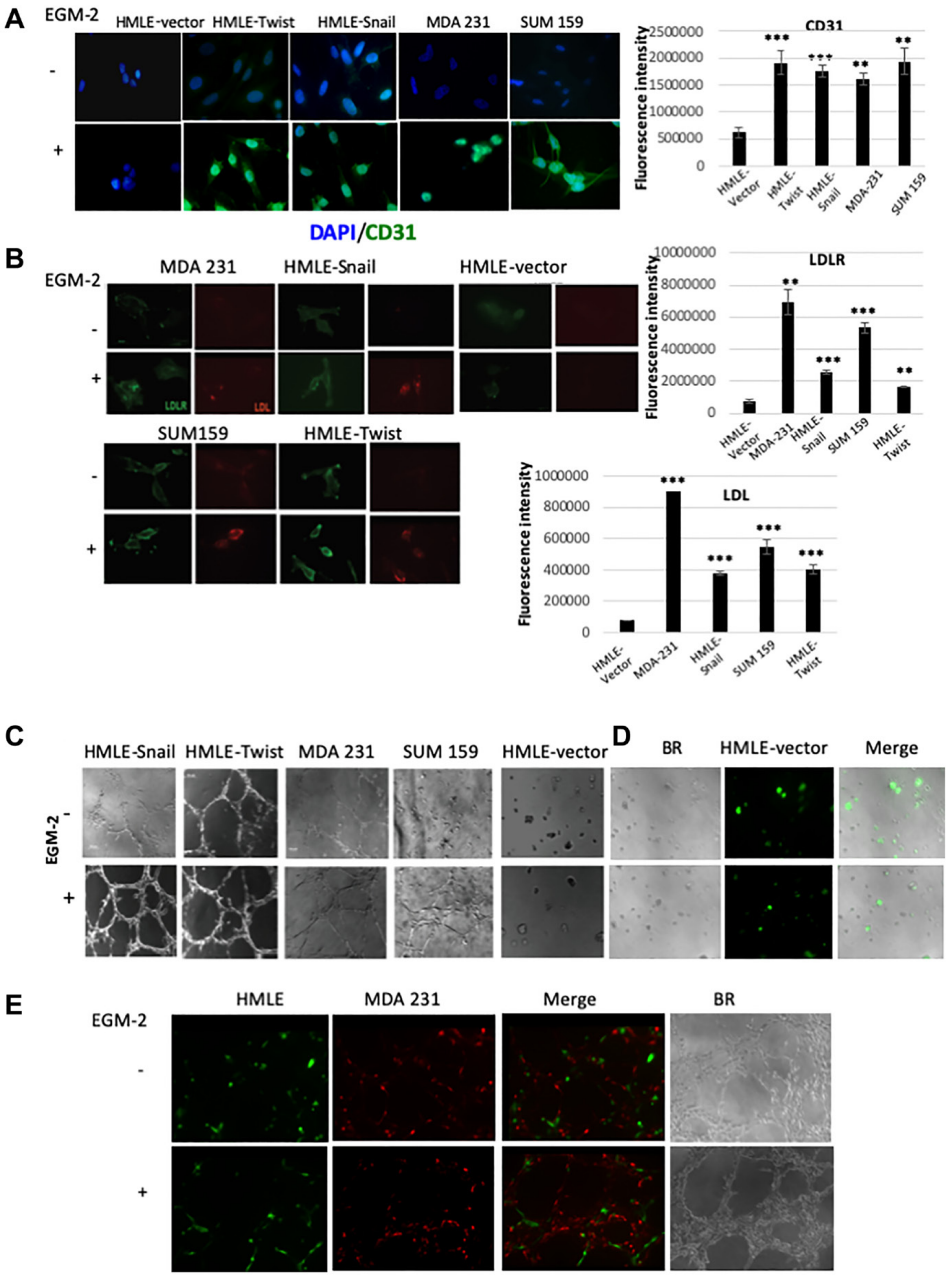
Cells that have undergone EMT can acquire endothelial-like phenotypic traits and functional behaviors *in vitro*. (**A**) The indicated cells were cultured in cell-specific medium (-EGM-2) or endothelial cell growth medium (+EGM-2). After 8–10 days, cells were fixed and immunostained for CD31 (green). Nuclei were counterstained with DAPI (blue). The bar graph on the right shows the mean fluorescence intensity of CD31 immunostaining in cells cultured in EGM-2 relative to cells cultured in cell-specific medium. (**B**) The indicated cells were cultured in cell-specific medium (-EGM-2) or endothelial cell growth medium (+EGM-2) for 2 days. Cells were incubated with an LDL-DyLight™ 549 conjugate for 4 hours prior to fixation and immunostaining. The distribution of the LDL-receptor (LDLR; green) and the LDL-DyLight™ 549 conjugate (LDL; red) were imaged using fluorescence microscopy. Note the punctate perinuclear staining pattern of the LDL-DyLight™ 549 conjugate, consistent with complex internalization and accumulation in lysosomal membranes. The mean fluorescence intensity of of LDL and LDLR in cells cultured in EGM-2, relative to cells cultured in cell-specific medium, is shown in the bar graphs to the right of the corresponding images. Scale bar: 20 μm (**C**) The indicated cells were cultured in cell-specific medium (-EGM-2) or endothelial cell growth medium (+EGM-2) and plated onto Matrigel. After 24 hours, the ability of the cells to organize into capillary-like structures was determined using brightfield microscopy. (**D**) GFP-labeled HMLE cells were plated onto Matrigel in cell-specific medium (-EGM-2) or endothelial cell growth medium (+EGM-2). After 24 hours, the cells were imaged using brightfield (BR) and fluorescence microscopy. Right panels are overlays of brightfield and fluorescent images (Merge). GFP-labeled HMLE cells (green) failed to form interconnecting tubular structures autonomously, irrespective of culture medium. (**E**) GFP-labeled HMLE (green) and RFP-labeled MDA-MB-231 (abbreviated to MDA 231; red) cells were co-mixed and plated onto Matrigel in the presence of EGM-2. After 24 hours, the cells were imaged using brightfield (BR) and fluorescence microscopy. Merged images of individual fluorescent channels show the incorporation of each cell type into mosaic tubular structures (Merge). Representative images are shown (*n* = 4). The fluorescence intensity for the evaluation of gene expression analysis was performed using ImageJ software [73, 74]. Student’s *t-test* was performed to test significance. Data indicate mean ± SEM. *n* = 5. ^*^ *P* < 0.05, ^**^ *P* < 0.01, ^***^ *P* < 0.001.

Given our findings above, we sought to determine whether cells that have undergone EMT exhibit the capacity to acquire phenotypic and functional characteristics of endothelial cells *in vitro*. In addition to HMLE-vector, HMLE-Snail, and HMLE-Twist cells, we employed two established breast cancer cell lines, namely MDA-MB-231 and SUM159 cells, representing the recently-recognized claudin-low intrinsic breast cancer subtype, which is enriched for mesenchymal features and cancer stem cell properties [52, 53]. For this, cells were cultured in endothelial cell growth medium, designated EGM-2, supplemented with VEGF, a potent pro-angiogenic stimulant [18]. After 8–10 days of culture in EGM-2, cells were fixed and stained for the pan-endothelial marker CD31. Consistent with the acquisition of an endothelial-like phenotype, EGM-2-cultured mesenchymal cells exhibited increased CD31 staining to differing degrees, compared with counterparts cultured in cell-specific medium. In HMLE-Twist, MDA-MB-231, and SUM159 cells, CD31 staining was mostly diffuse and nuclear, cytosolic with variably-sized CD31-positive puncta (Figure 5A). Interestingly, in addition to a nuclear/cytosolic/membranous staining pattern, EGM-2-cultured cells (except for HMLE-vector cells) exhibited strong nuclear CD31 staining, which has also been observed e.g., in pancreatic tumors [54]. For the most part, CD31 was not expressed in undifferentiated mesenchymal cells cultured in cell-specific medium. However, weak CD31 staining was detected in the cytosol and occasional nuclei of undifferentiated HMLE-Twist and HMLE-Snail cells, suggesting that the Snail and Twist EMT programs confer basal CD31 expression, even in the absence of an angiogenic stimulus. This is reminiscent of previously reported findings that Twist overexpression increased CD31 protein levels in a subpopulation of head and neck cancer cells [55].

We also evaluated whether EGM-2-cultured mesenchymal cells exhibit functional behaviors associated with the mature endothelial phenotype. We, therefore, examined the internalization of Low-Density Lipoprotein (LDL)—the major carrier of cholesterol in the blood—through receptor-mediated endocytosis, which comprises a well-established, physiologically-relevant, functional assay for the endothelial lineage [56]. This assay measures the internalization of LDL complexes, via the so-called “scavenger pathway”, and their incorporation into lysosomal membranes, modeling accumulation of cholesterol and the formation of atherosclerotic plaques in arterial endothelial cells [59]. Using immunofluorescence, we found that undifferentiated HMLE-Snail and HMLE-Twist cells, as well as established mesenchymal breast cancer cells, exhibited a cytoplasmic/membranous staining pattern for the LDL-receptor (LDLR), compared with the weak and mostly diffuse staining of vector-transduced epithelial counterparts (HMLE-vector; Figure 5B). In addition, we detected an increased staining intensity for LDLR in EGM-2-cultured mesenchymal cells (Figure 5B). The observed granular staining pattern of LDLR, at sites close or at the plasma membrane, is consistent with the well-documented constitutive endocytic trafficking of LDLR via clathrin-coated pits [57].

We next examined the ability of cells, cultured in cell-specific medium or EGM-2, to internalize fluorophore-labeled LDL. Notably, only EGM-2-cultured mesenchymal cells exhibited significant uptake of fluorescently-tagged LDL, with the internalized complex accumulating in lysosomal membranes, as indicated by the predominantly punctate perinuclear staining pattern (Figure 5B) [58]. Undifferentiated mesenchymal HMLE-Snail, HMLE-Twist, and MDA-MB-231 cells exhibited only minimal LDL-uptake, barely above the low background fluorescence levels (Figure 5B). However, low-intensity staining—indicative of basal LDL-uptake activity—was manifest in SUM159 cells, cultured in cell-specific medium (Figure 5B). This suggests that SUM159 cells may be primed towards certain aspects of endothelial functionality, although they appear devoid of basal CD31 levels. As expected, epithelial HMLE-vector cells remained unstained indicating a failure to acquire the capacity to internalize LDL in EGM-2 medium, thus confirming that differentiated epithelial cells lack inherent cellular plasticity.

Consistent with the acquisition of an endothelial-like functionality, EGM2-cultured mesenchymal cells also exhibited the ability to organize into capillary-like structures, when plated onto basement membrane-like growth factor-reduced Matrigel, rapidly forming an interconnected polygonal network within 6 hours (Figure 5C). Indeed, we observed a significant increase in tube-like structure formation in EGM-2-cultured mesenchymal cells, as compared with counterparts grown in cell-specific medium. Nevertheless, HMLE-Snail and MDA-MB-231 cells, plated onto Matrigel and cultured in cell-specific medium, exhibited a pronounced elongated cell shape and formed discontinuous tubules that, however, stopped short of generating three-dimensional capillary-like structures (3D-capillary-like network) (Figure 5C). Moreover, HMLE-Twist cells appeared to exhibit vasculogenic activity, even in the absence of EGM-2, with the corresponding growth patterns suggestive of pre-differentiation. These behaviors were not shared by epithelial HMLE-vector cells, irrespective of culture medium, or undifferentiated SUM159 cells, both of which failed to migrate and/or survive under these conditions (Figure 5C).

Our *in vivo* studies raise the possibility that the emergence of cells that have undergone EMT, in the tumor niche, may influence the proclivity of epithelial tumor cells to integrate into the nascent tumor vasculature (Figure 4C). In order to test whether cells that have undergone EMT can influence the behavior of epithelial cells in their vicinity, we employed a co-culture model in combination with the Matrigel tube-formation assay. For this, we transduced epithelial HMLE and mesenchymal MDA-MB-231 cells with expression constructs encoding GFP and RFP respectively, and plated these cells onto Matrigel in the presence of cell-specific medium or EGM-2. As expected, HMLE cells failed to form interconnected tubular structures autonomously (Figure 5D), consistent with the fact that HMLE cells lack intrinsic migratory capacity and do not acquire endothelial characteristics following culture in EGM-2 *in vitro* (Figure 5D). However, when GFP-labeled HMLE cells were co-cultured in the presence of RFP-labeled MDA-MB-231 cells, the admixtures organized into tubular structures bearing both fluorescent labels. We also performed the converse experiment, using RFP-labeled HMLE and GFP-labeled MDA-MB-231 cells, which generated similar mosaic tubular structures (Figure 5E). These results are consistent with the notion that MDA-MB-231 cells can promote the integration of epithelial HMLE cells into nascent tubular networks. Collectively, our *in vitro* findings suggest that, under the appropriate culture conditions, cells that have undergone EMT or established mesenchymal breast cancer cell lines can acquire endothelial-like phenotypic traits and functional behaviors *in vitro*, as well as modulate the vasculogenic behavior of differentiated epithelial cells. Furthermore, mesenchymal-like cells, or subpopulations thereof, may harbor some endothelial traits at baseline, predisposing them to differentiation along the endothelial lineage.

### FOXC2 plays a central role in regulating endothelial differentiation potential *in vitro* and *in vivo*

We and others have previously shown that the transcription factor FOXC2 functions downstream of multiple EMT-inducing stimuli and that FOXC2 knockdown abrogates EMT-associated stem-like properties [27–29]. In addition, we report herein that FOXC2 expression is induced in the hypoxic tumor cores, commensurate with the induction of EMT. Taken together with the known functions of FOXC2 in the regulation of physiological and tumor angiogenesis [34–36], we hypothesized that FOXC2 may play a central role in the capacity of cells that have undergone EMT to acquire an endothelial phenotype.

**Figure 6:**
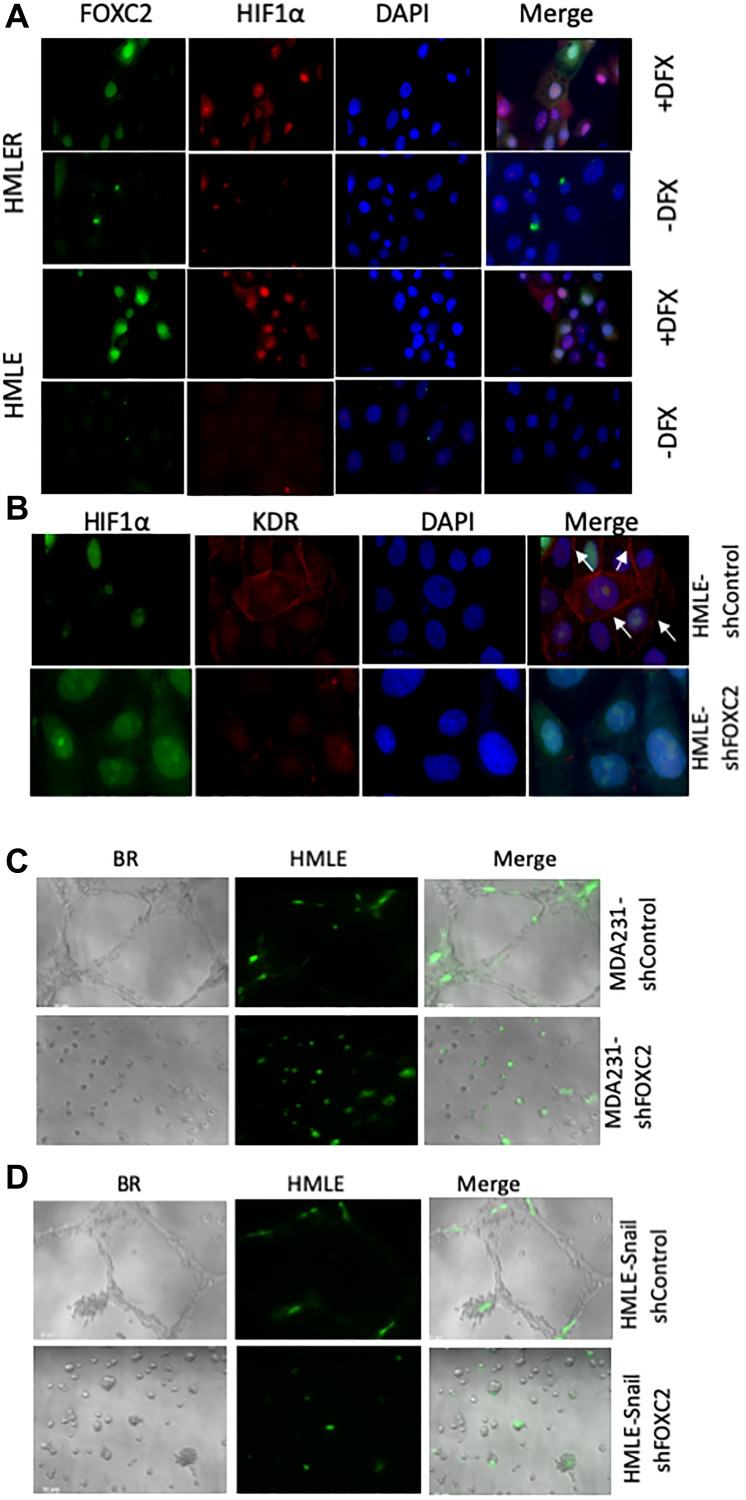
FOXC2 is necessary for the acquisition of endothelial phenotypic and functional characteristics *in vitro*. (**A**) Immortalized (HMLE) and RAS-transformed (HMLER) human mammary epithelial cells were plated in cell-specific medium. After 24 hours, the cells were treated either with vehicle or desferrioxamine (DFX), a hypoxia-mimetic, for 48 hours prior to fixation and immunostaining for FOXC2 (green) and HIF-1α (red). Nuclei were counterstained with DAPI (blue). Right panels are merged images of individual channels. Scale bar: 20 μm. (**B**) HMLE cells, transduced with shRNAs targeting firefly luciferase (shControl) or FOXC2 (shFOXC2), were treated with DFX for 48 hours. Following DFX treatment, the cells were fixed and immunostained for HIF-1α (green) and KDR (red). Nuclei were counterstained with DAPI (blue). Right panels are merged images of individual channels. Scale bar: 20 μm (**C**) GFP-labeled HMLE cells (green) were co-mixed with either MDA-MB-231-shControl or MDA-MB-231-shFOXC2 cells and plated onto Matrigel in EGM-2. After 24 hours, the cells were imaged using brightfield (BR) and fluorescence microscopy. Right panels are overlays of brightfield and fluorescent images. The impact of FOXC2 knockdown on the ability of MDA-MB-231 cells to form vascular-like networks and stimulate the incorporation of GFP-labeled HMLE cells into mosaic structures was examined. (**D**) GFP-labeled HMLE cells (green) were co-mixed with either HMLE-Snail-shControl or HMLE-Snail-shFOXC2 cells and plated onto Matrigel in EGM-2. After 24 hours, the cells were imaged, using brightfield (BR) and fluorescence microscopy, and the formation of tubular structures assessed. Right panels are overlays of brightfield and fluorescent images. Representative images are shown. MDA-MB-231 have been abbreviated to MDA 231.

As we observed upregulation of FOXC2 expression in hypoxic tumor cores, we first examined the expression of FOXC2 and HIF-1α in immortalized (HMLE) and RAS-transformed (HMLER) human mammary epithelial cells, following exposure to DFX, an iron chelator known to function as a hypoxia-mimetic. We observed that DFX-treated HMLE and HMLER cells exhibited intense nuclear co-staining for FOXC2 and HIF-1α, compared with a distinct lack of nuclear staining in vehicle-treated counterparts, which exhibited only occasional HIF-1α- or FOXC2-positive juxtanuclear puncta (Figure 6A). These results are consistent with the induction of FOXC2 expression and its nuclear translocation in response to hypoxia *in vitro*, in keeping with the expression pattern of FOXC2 in the hypoxic tumor cores *in vivo* (Figure 2B). However, following DFX treatment *in vitro*, we detected HIF-1α in the nuclear compartment, whereas *in vivo* we mostly observed HIF-1α punctate cytoplasmic staining, as discussed previously. The reasons for this discrepancy are unclear, but the respective HIF-1α staining patterns may reflect differences in the pathways engaged under physiological hypoxia and the DFX-model, or cell-specific differences. The elevated protein levels of FOXC2 and HIF-1α in DFX-treated cells, compared with vehicle-treated counterparts, were confirmed by immunoblotting (Supplementary Figure 2).

We next treated HMLE cells, transduced with shRNAs targeting firefly luciferase (shControl) or FOXC2 (shFOXC2) [28, 29], with DFX. Following DFX treatment for 48 hours, HIF-1α was detected in the nuclei of both HMLE-shControl and HMLE-shFOXC2 cells, indicating that hypoxia-induced HIF-1α accumulation proceeds independently of FOXC2 (Figure 6B). In this model, we also examined the expression of kinase insert domain receptor (KDR)—also known as vascular endothelial growth factor receptor 2 (VEGFR-2) or fetal liver kinase 1 (FLK1)—a well-established marker of the endothelial lineage that promotes cell proliferation and survival in response to its pro-angiogenic ligand, VEGF. Interestingly, we found that FOXC2 shRNA-mediated knockdown elicited a clear reduction in the membrane localization of KDR without significantly affecting nuclear KDR levels (Figure 6B), perhaps suggesting that FOXC2 knockdown differentially impacts external and internal VEGF/KDR signaling loops [59].

We and others have previously shown that FOXC2 knockdown compromises the EMT phenotype [25, 27–29]. Accordingly, depletion of FOXC2 in MDA-MB-231 and HMLE-Snail cells restored the expression of E-cadherin and led to a downregulation of the levels of vimentin (Supplementary Figure 3). To further test whether FOXC2 knockdown compromises the manifestation of endothelial-like functional behaviors *in vitro*, we employed our co-culture model in combination with the Matrigel tube-formation assay. For this, we co-mixed GFP-labeled HMLE cells with MDA-MB-231 or HMLE-Snail cells, transduced with shRNA targeting either firefly luciferase (MDA-MB-231-shControl, HMLE-Snail-shControl) or FOXC2 (MDA-MB-231-shFOXC2, HMLE-Snail-shFOXC2). In this assay, MDA-MB-231-shControl and HMLE-Snail-shControl cells readily formed a distinctive polygonal tubular network and potentiated the incorporation of GFP-labeled HMLE cells into the resulting tubular structures (Figure 6C and 6D). Strikingly, FOXC2 knockdown abrogated the tube-forming capacity of MDA-MB-231 and HMLE-Snail cells alike. This observation is consistent with our previous findings that FOXC2 knockdown compromises the inherent migratory potential of mesenchymal cells [28]. Our data suggest that FOXC2 knockdown renders MDA-MB-231 and HMLE-Snail cells inherently unable to migrate to form tubular structures in Matrigel and concurrently inhibits their ability to influence the tube-forming behavior of admixed HMLE cells (Figure 6C and 6D). Together, these findings point to an important role for FOXC2 in the regulation of endothelial differentiation potential *in vitro*.

**Figure 7:**
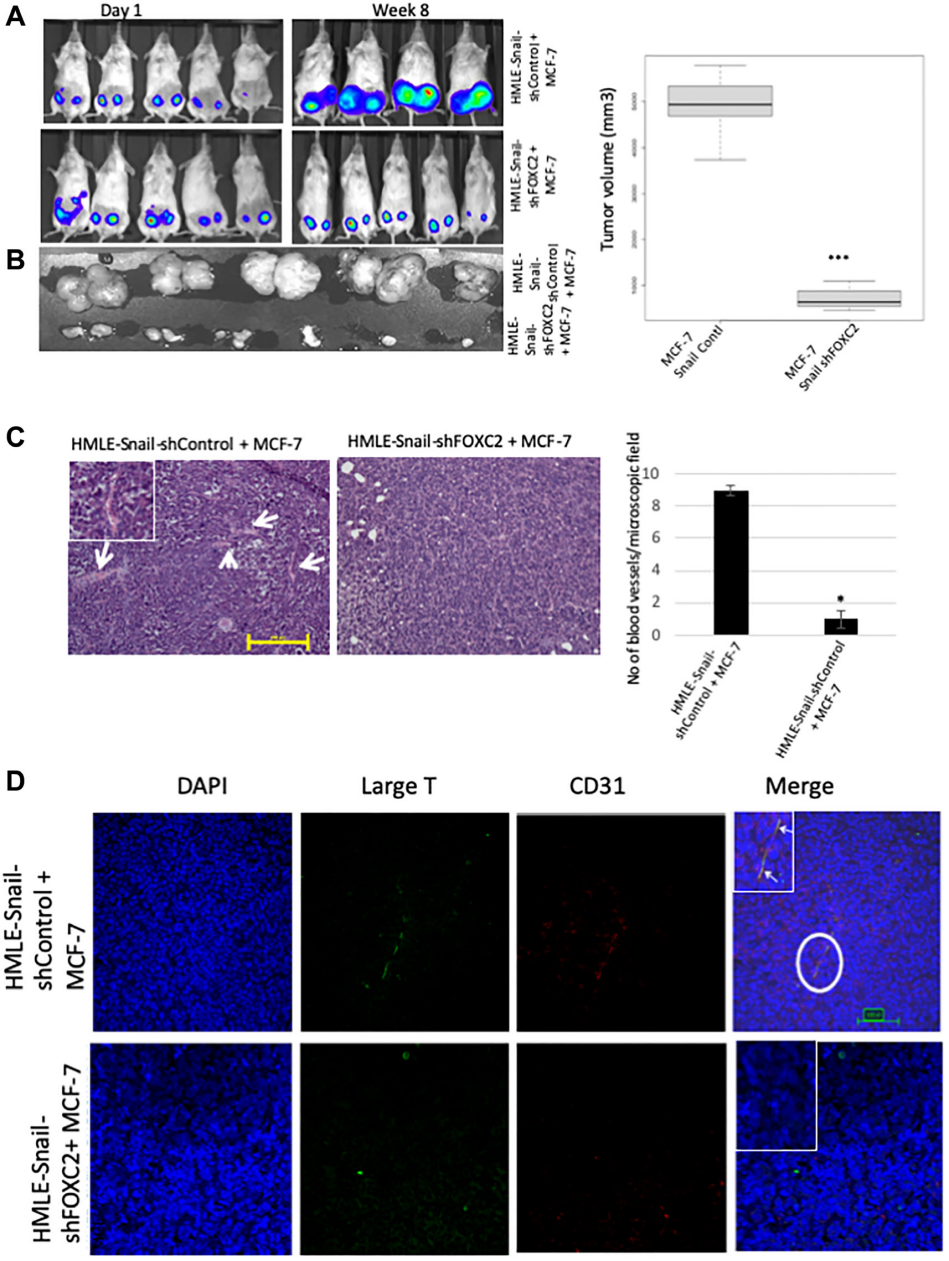
FOXC2 is necessary for the ability of cells that have undergone EMT to augment neoangiogenesis. (**A**) RFP/luciferase-labeled MCF-7 cells were admixed with either HMLE-Snail-shControl or HMLE-Snail-shFOXC2 cells, and orthotopically implanted into female NOD/SCID mice. The bioluminescent signal emitted by the implanted cells, or the resulting primary tumors, was recorded using bioluminescent imaging 1 day post injection and 8 weeks post implantation, respectively. Blue, least intense, to red, most intense. (**B**) The tumors from the mice in (A) were excised and imaged prior to processing for histology. Graphical representation of the tumor volumes at endpoint is shown on the right. (**C**) Tissue sections, from the core regions of the tumors in (B), were stained with H&E. Arrows indicate the location of blood vessels. Scale bar, 100 μm. The average number of blood vessels per microscopic field is plotted on the right. (**D**) Tissue sections, from the core regions of the tumors in (B), were co-stained with antibodies directed against the SV40 large-T antigen (green) and human CD31 (red). Nuclei were counterstained with DAPI (blue). Right panels are merged images of individual channels. Insets represent high-magnification images of encircled areas. Arrows indicate SV40 large-T antigen/CD31 double-positive cells. Scale bar, 100 μm. *n* = 5 mice/group. Representative images are shown.

In order to ascertain whether FOXC2 is necessary for the ability of cells that have undergone EMT to augment tumor growth and angiogenesis *in vivo*, we co-mixed HMLE-Snail-shControl or HMLE-Snail-shFOXC2 cells with RFP/luciferase-labeled MCF-7 cells. As before, the admixtures were orthotopically implanted into the contralateral pair of fourth mammary fat-pads of female NOD/SCID mice. At 8 weeks post implantation, the mice were sacrificed, and the tumors were excised, imaged, and processed for histology. As expected, admixtures of MCF-7 and HMLE-Snail-shControl cells generated tumors of a considerable size/volume as evidenced by the intense bioluminescent signal, detected in the tumor-bearing mice, and the corresponding macroscopic images of the excised primary tumors (Figure 7A and 7B). In stark contrast, FOXC2 knockdown abrogated the ability of HMLE-Snail cells to potentiate MCF-7 cell growth *in vivo,* yielding markedly smaller tumors (Figure 7A and 7B). Accordingly, H&E staining showed that the small tumors, formed by admixed MCF-7 and HMLE-Snail-shFOXC2 cells, lacked discernible blood vessels, markedly contrasting with the extensive vascularization exhibited by tumors established from MCF-7/HMLE-Snail-shControl admixtures (Figure 7C). Using immunofluorescence, we detected SV40 large-T/CD31 double-positive cells within the vascular networks of tumors formed by admixed MCF-7/HMLE-Snail-shControl cells, confirming the descendancy of CD31-positive cells from HMLE-Snail-shControl cells (Figure 7D). As expected, we did not observe SV40 large-T/CD31 double-positive cells in tumors formed by admixed MCF-7 and HMLE-Snail-shFOXC2 cells (Figure 7D). These data suggest that FOXC2 is necessary for the ability of cells that have undergone Snail-induced EMT to acquire CD31 expression *in vivo*, and contribute to the development of intra-tumoral vessels. Taken together, these findings indicate that FOXC2 plays a central role in regulating the endothelial differentiation potential of cells that have undergone EMT *in vitro* and *in vivo*.

## DISCUSSION

Previous studies demonstrated that the induction of EMT bestows stem-like capabilities upon differentiated epithelial carcinoma cells, exemplified by the acquisition of enhanced tumor-initiating potential and the capacity to differentiate into multiple mesodermal lineages *in vitro* [25, 26]. A recent study showed that miR-151a enhances angiogenesis in normal lung tissue and non-small cell lung cancer 3D tumor spheroids by increasing the levels of the EMT-inducing transcription factor Slug [60]. However, a direct connection between the induction of EMT and the onset of angiogenesis has not yet been established *in vivo*. In the present study, we demonstrate that cells that have undergone EMT can promote tumor growth and neovascularization either indirectly, by promoting endothelial transdifferentiation of carcinoma cells in the tumor milieu, or directly, through the acquisition of an endothelial-like phenotype, with the central EMT-mediator FOXC2 playing a key role in these processes.

We first established a tumor model whereby mice, orthotopically implanted with MCF-7 breast epithelial adenocarcinoma cells, were sacrificed at different timepoints post implantation, yielding tumors of different sizes. Using this model, we found that the patterns of hypoxia and EMT are related to the degree of vascularization in tumors of different sizes. Indeed, tumors sized ≤ 2 mm were markedly hypo-vascularized compared with the larger tumors, which displayed progressively developed vascular networks. Only occasional cells in the tumors sized ≤ 2 mm exhibited cytoplasmic HIF-1α-positive puncta, likely reflecting the early stages of HIF-1α stabilization in localized areas of low oxygen tension. Whereas the cores of the tumors sized 5–7 mm displayed pronounced HIF-1α staining, demarcating hypoxic regions, tumors sized 14–15 mm exhibited a heterogeneous, more diffuse, and somewhat weaker HIF-1α staining pattern, suggesting that the establishment of a vascular network partially ameliorated the oxygen deficit. Therefore, these tumors of differing sizes broadly represent the progression from poorly-vascularized hypoxic tumors to well-vascularized adequately-oxygenated counterparts.

Most interestingly, the blood vessels of the larger tumors, sized 5–7 mm or 14–15 mm, were decorated by a human-specific anti-CD31 antibody, suggesting that neoangiogenesis in these tumor cores entailed MCF-7 cell transdifferentiation towards an endothelial phenotype. These results are consistent with studies suggesting that a range of tumor cell types can assume an endothelial-like phenotype *in vivo* via transdifferentiation [12–16]. As we used a human-specific CD31 antibody, our data do not preclude the recruitment of host endothelial progenitor cells or host-vessel co-option as additional mechanisms contributing to the establishment of the tumor vasculature. However, the contiguity of the observed CD31-staining patterns suggests that many of the intra-tumoral vessels, in this model, are predominantly of human tumor cell origin rather than mouse/human mosaics.

Immunofluorescent staining of this series of MCF-7 tumors, allowed to grow to different sizes, also suggests that tumor outgrowth is accompanied by the initiation of EMT events within the hypoxic tumor core. Accordingly, we detected increased expression of the mesenchymal marker vimentin and the EMT-inducing transcription factor FOXC2 in HIF-1α-positive regions, concomitant with greatly diminished E-cadherin membrane staining at discrete foci, likely denoting regional loss of cell-cell cohesion and local invasion. Of note, FOXC2 is not a direct transcriptional repressor of the *CDH1* gene encoding E-cadherin, unlike most other EMT-inducing transcription factors [27]. However, FOXC2 has been shown to repress p120-catenin, known to tether the E-cadherin complex to the cell membrane, leading to destabilization of the E-cadherin complex [61]. In fact, FOXC2 activity is considered to be mostly associated with the induction of mesenchymal traits rather than the repression of epithelial features [27]. Indeed, we observed markedly increased vimentin staining, ostensibly mirroring the extent of FOXC2 immunostaining. These data also suggest that vimentin upregulation is an early event in tumor progression that precedes loss of E-cadherin from the cell membrane. Overall, our findings demonstrate that as MCF-7 tumors outgrow their blood supply and develop hypoxic regions, cells exhibit phenotypic changes consistent with the initiation of EMT as well as transdifferentiation to a CD31-positive endothelial-like lineage.

To better understand the role of hypoxia-induced EMT in the continued outgrowth and neovascularization of epithelial tumors, we next analyzed tumors established by orthotopic implantation of admixtures of RFP/luciferase-labeled MCF-7 cells and cells experimentally induced to undergo EMT *in vitro*. Using this tumor model, we made the striking observation that co-implantation of MCF-7 cells, with either HMLE-Snail or HMLE-Twist cells, yielded highly-vascularized tumors of significantly greater volume, compared with tumors formed by MCF-7 cells injected either alone or co-mixed with epithelial HMLE-vector cells. As expected, intra-tumoral vessels contained RFP/CD31 double-positive cells, consistent with MCF-7 endothelial transdifferentiation, similar to what we observed with the larger MCF-7 tumors sized 5–7 mm or 14–15 mm. Strikingly, the incidence of these CD31/RFP double-positive cells was most prevalent in MCF-7/HMLE-Snail tumors, with only occasional CD31-positive cells detected in MCF-7/HMLE-vector tumors, suggesting that mesenchymal HMLE-Snail cells promote the transdifferentiation of MCF-7 cells towards an endothelial phenotype. Indeed, our co-culture tube-formation assays support this conclusion. Accordingly, co-mixing HMLE cells with mesenchymal MDA-MB-231 or HMLE-Snail cells elicited the incorporation of GFP-labeled HMLE cells into capillary-like structures, suggesting that cells that have undergone EMT can influence the behavior of neighboring epithelial cells in the tumor milieu. These properties are exclusively associated with the mesenchymal phenotype since: 1) HMLE-vector cells were unable to potentiate the tumor growth of admixed MCF-7 cells *in vivo*, 2) HMLE-vector cells failed to acquire endothelial markers when cultured in EGM-2 *in vitro*, and 3) GFP-labeled HMLE cells lacked the ability to autonomously organize into tubular structures in Matrigel, but were readily incorporated into tubular structures upon co-culturing with mesenchymal cells. These findings are consistent with the fact that epithelial cells lack inherent cellular plasticity and intrinsic migratory potential. Most importantly, these data collectively suggest that the presence of cells that have undergone EMT in the tumor milieu can promote carcinoma cell transdifferentiation towards an endothelial-like lineage presumably via paracrine signaling or changes in extracellular matrix deposition.

Notably, vessels in the MCF-7/HMLE-Snail tumor cores also contained cells that co-stained for CD31 and SV40 large-T antigen, consistent with *in vivo* differentiation of HMLE-Snail cells into endothelial-like counterparts, competent for integration into nascent vascular structures. Interestingly, in human tissue specimens of poorly differentiated, invasive ductal carcinomas of the breast and their associated lymph node metastases, a subpopulation of tumor cells has been shown to exhibit dual staining for CD31 and CD44, the stem-cell associated antigen acquired by epithelial cells induced to undergo EMT [62]. Together, these results suggest that the induction of EMT is associated with the acquisition of CD31 positivity both in our tumor models and human breast tumor specimens.

We did not detect CD31/SV40 large-T double-positive cells in the MCF-7/HMLE-vector tumors, but we did observe rare RFP-negative cells staining positive for human CD31 in tumors formed by MCF-7/HMLE-vector admixtures, which we presume would stain for SV40 large-T antigen (i.e., are derived from HMLE-vector cells). Therefore, the rare CD31-positive cells, observed in MCF-7/HMLE-vector tumors, may derive from either epithelial cell input through transdifferentiation and likely represent endothelial differentiation events early in neoangiogenesis. We further postulate that the rarity of CD31-positive cells in either the ≤ 2 mm-sized MCF-7 tumors or the MCF-7/HMLE-vector tumors is attributable to the fact that, at the time of harvesting, these tumors had not reached the critical tumor size threshold for hypoxia to evoke induction of EMT and/or endothelial transdifferentiation in MCF-7 or HMLE-vector cells.

The mechanisms underpinning new vessel formation, in our tumor models, involve phenotypic conversion of cells that have undergone EMT into CD31-positive cells. Importantly, this is distinct from vasculogenic mimicry, whereby tumor cells form “fluid-conducting networks” without, however, acquiring endothelial cell markers, such as CD31 [10, 11]. Although some studies have linked the EMT-inducing transcription factors Twist and ZEB1 to vasculogenic mimicry [63–65], or Slug to angiogenesis [60], our data are consistent with a mechanism akin to EMT-associated dedifferentiation conferring enhanced cellular plasticity and endothelial differentiation potential. In support of this idea, cells that have undergone EMT acquire phenotypic markers of mature endothelial cells, notably the expression of CD31 and KDR, following culture in EGM-2. Moreover, these cells also gain functional attributes of endothelial cells such as the ability to internalize LDL and organize into interconnected capillary-like structures in Matrigel. However, from our phenotypic and functional assays, it appears that subpopulations of mesenchymal cells are intrinsically adorned with some endothelial traits, to differing degrees, suggesting that these cells may be partially primed towards an endothelial phenotype. Similarly, Twist overexpression increased CD31 protein levels in a subpopulation of head and neck cancer cells in the absence of angiogenic stimuli [58]. Interestingly, the highly tumorigenic, mesenchymal-like, sphere-forming subpopulations of luminal (MCF-7) and basal (MCF10AT, MCF10DCIS) breast cancer cell lines have recently been shown to express endothelial markers and organize into CD31-positive tubular structures in response to nutrient deprivation, a potent pro-angiogenic stimulus [66]. Together, these findings are consistent with the notion that cells that have undergone EMT, claudin-low breast cancer cells, or tumorigenic subpopulations of luminal and basal tumor types may respond to extracellular cues, emanating from the tumor microenvironment, by upregulating and/or reinforcing endothelial traits. This phenomenon likely enables adaptation of tumor cells to the adverse conditions that prevail in the hostile tumor microenvironment.

Our *in vitro* studies show that DFX-treatment of HMLE cells is accompanied by the upregulation of HIF-1α, FOXC2, and KDR, which serve as indicators of the hypoxic response, the induction of EMT, and a shift to an endothelial phenotype, respectively. This supports the links between hypoxia, EMT-associated plasticity, and the acquisition of endothelial traits. In tumors, endothelial transdifferentiation may be initiated and/or potentiated through the engagement of KDR by elevated VEGF levels in the tumor microenvironment. In fact, the EMT-associated upregulation of VEGF-A has been recently linked to increased tumor angiogenesis [67]. However, VEGF-A overexpression alone is not sufficient to augment tumor angiogenesis and requires additional angiogenic mediators such as, presumably, KDR [67, 68]. Indeed, while hypoxia is associated with nuclear accumulation of mostly unphosphorylated KDR forms in many human neoplastic cell lines, only concurrent exposure to hypoxia and VEGF elicits a marked increase in phosphorylated/activated KDR levels [67]. Interestingly, we found that FOXC2 knockdown in DFX-treated HMLE cells attenuated the membrane localization of KDR, relative to vehicle-treated cells, without significantly affecting nuclear KDR levels. This raises the possibility that FOXC2 regulates the external VEGF/KDR autocrine loop both by modulating cell surface-associated KDR levels and by promoting the expression of secreted VEGF-A [31]. The mechanisms whereby FOXC2 influences the external VEGF/KDR autocrine loop, and the role of nuclear KDR following FOXC2 knockdown, warrant further investigation.

Having established that hypoxic core regions exhibit nuclear FOXC2 immunostaining and that FOXC2 knockdown compromises the cell surface-associated expression of the endothelial marker KDR, we next determined whether FOXC2 is necessary for the capacity of cells that have undergone EMT to exhibit endothelial-like behaviors. Consistent with a vital role for FOXC2 in the regulation of endothelial differentiation potential, FOXC2 knockdown abolished the inherent tube-forming ability of MDA-MB-231 and HMLE-Snail cells as well as their ability to promote the incorporation of HMLE cells into tubular networks *in vitro*. Most importantly, FOXC2 knockdown abrogated the potentiating effect of HMLE-Snail cells on the growth and vascularization of admixed MCF-7 cells *in vivo*, with MCF-7/HMLE-Snail-shFOXC2 admixtures yielding markedly smaller tumors compared with co-mixed MCF-7 and HMLE-Snail-shControl cells. These considerations underscore the interplay of microenvironmental cues and cell-intrinsic factors in endothelial transdifferentiation and suggest that FOXC2 plays an important role in these processes.

Overall, our findings are consistent with the notion that the phenotypic attributes of cells within growing tumors are eminently pliable and that, as tumor size and the oxygen deficit increase, carcinoma cells become progressively dedifferentiated towards a mesenchymal, stem-like phenotype. Indeed, we have previously used mathematical modeling to show that exposure to hypoxia augments the rate of dedifferentiation of non-stem, differentiated epithelial cells resulting in a shift towards a stem-like, plastic phenotype with increased EMT features [69]. On the basis of our findings herein, we propose that the induction of EMT contributes to tumor neoangiogenesis in two different ways: Firstly, indirectly, by modifying the tumor niche, through paracrine signaling or alterations in extracellular matrix deposition, to promote the endothelial transdifferentiation of epithelial tumor cells. Secondly, by generating stem-like cells that can assume endothelial-like phenotypic and functional attributes and directly integrate into the nascent tumor vasculature. We also report that FOXC2, a common denominator of multiple EMT pathways, plays a vital role in the acquisition of endothelial phenotypic and functional characteristics *in vitro* and *in vivo*, with the corollary that inhibition of FOXC2 signaling may compromise tumor neoangiogenesis.

## MATERIALS AND METHODS

### Cell culture

Immortalized human mammary epithelial (HMLE) cells expressing empty vector pWZL (HMLE-vector), Snail (HMLE-Snail) or Twist (HMLE-Twist), and RAS-transformed HMLE (HMLER) cells were maintained as previously described [37, 51]. HMLE cells were transduced, as previously described [39], with expression constructs encoding green- or red-fluorescent protein (pMIG or RFP/luciferase, respectively), for use in tube-formation assays. MCF-7, MDA-MB-231, and SUM159 human breast cancer cells were cultured in cell-specific medium as previously detailed [28]. HMLE-Snail or MDA-MB-231 cells, transduced with shRNA targeting either firefly luciferase (shControl) or FOXC2 (shFOXC2), were previously described [28, 29].

Cells were treated with vehicle (dH2O) or 100 μM desferrioxamine (DFX) for 24 hours prior to processing for immunofluorescence. For the induction of endothelial differentiation, cells were seeded at a density of 6000 cells/well and cultured for 8–10 days in endothelial cell growth medium (EGM-2 BulletKit™, CC-3162; Lonza Inc, Rockland ME, USA), supplemented with 2% fetal bovine serum and VEGF (designated EGM-2), as previously described [62].

### LDL-uptake assay

HMLE, HMLE-Snail, HMLE-Twist, MDA-MB-231, and SUM 159 cells were cultured at a density of 3 × 10^4^ cells/well in a 96-well plate for 2 days in either cell-specific medium or EGM-2. Human Low-Density Lipoprotein (LDL) conjugated to DyLight™ 549 (10 μg/ml)—an orange-to-red fluorescent probe enabling detection of LDL-uptake—was added to the cultured cells. Four hours after LDL-DyLight™ 549 addition, cells were fixed and stained for LDL-receptor using a rabbit anti-LDL-receptor primary antibody and DyLight™ 488-conjugated secondary antibody, according to the manufacturer’s instructions (Cell-Based LDL-Uptake Assay Kit, ab133127; Abcam, Cambridge, MA, USA). The distribution patterns of LDL-receptor, and the uptake of the LDL-DyLight™ 549 conjugate were imaged using fluorescence microscopy. All images were acquired using an inverted Zeiss Axio Observer fluorescent microscope.

### Endothelial cell tube-formation assay

In order to evaluate the formation of capillary-like structures upon plating onto Matrigel, 24-well plates were coated with 250 μl of chilled Matrigel matrix (354234; Corning Incorporated-Life Sciences, Tewksbury, MA, USA). Cells were seeded at a density of 6000 cells/well on Matrigel-coated 24-well tissue culture plates, and cultured in the presence of either cell-specific medium or EGM-2, in a humidified incubator at 5% CO2 for 6–24 hours. The formation of capillary-like structures was observed over time and images were acquired at 6 and 24 hours post plating. All images were acquired using an inverted Zeiss Axio Observer fluorescent microscope.

### Imunofluorescence and antibodies

Immunofluorescent staining of cells and tumor sections was performed as previously described [70, 71]. The primary antibodies, employed in this study, were raised against the following antigens: HIF-1α (ab51608; Abcam), FOXC2 (rat anti-mouse FOXC2 antibody, which also cross-reacts with human FOXC2; developed by Dr. Naoyuki Miura, Hamamatsu University School of Medicine, Japan), E-cadherin (61081; BD Biosciences, San Jose, CA, USA), vimentin (MA1-19319; Thermo Fisher Scientific, Asheville, NC, USA), CD31 (ab28364; Abcam), KDR (MA5-15556; Novus, Centennial, CO, USA), RFP (ab62341; Abcam), SV40 large-T antigen (sc-47; Santa Cruz Biotechnology Inc., Dallas, TX, USA). Fluorescently-labeled secondary antibodies were from Thermo Fisher Scientific.

### Animal studies

Non-obese diabetic/severe combined immunodeficient (NOD/SCID) mice were purchased from the Jackson Laboratory (Bar Harbor, ME, USA). All mouse procedures were approved by the Animal Care and Use Committee of the University of Texas MD Anderson Cancer Center and performed in accordance with Institutional policies. To examine primary tumor formation and generate tumors of differing sizes, 1 × 10^6^ RFP/luciferase-labeled MCF-7 cells were injected into the contralateral pair of the fourth inguinal mammary fat-pads of female NOD/SCID mice. The mice were monitored for the emergence of palpable tumors, measuring approximately ≤ 2 mm, 5–7 mm, and 14–15 mm in longitudinal diameter (corresponding to 2, 6, and 10 weeks post implantation, respectively). At appropriate time intervals, the mice were sacrificed, and tumors of different sizes were harvested. To assess the ability of cells that have undergone EMT to potentiate tumor growth of admixed MCF-7 cells, 0.5 × 10^6^ RFP/luciferase-labeled MCF-7 cells were admixed with 0.5 × 10^6^ MCF-7, HMLE-vector, HMLE-Snail, or HMLE-Twist cells, and co-injected into the contralateral pair of fourth mammary fat-pads of female NOD/SCID mice. For these experiments, mice were sacrificed 10 weeks post implantation. All mice were assessed weekly for tumor growth both by palpation and bioluminescent imaging (data not shown). In order to calculate the tumor volume, the longitudinal (length) and transverse (width) diameters were determined using external caliper measurements. Tumor volumes were calculated using the modified ellipsoid formula: Tumor volume = 1/2 (length × width^2^) [72]. For bioluminescent imaging of developing tumors, mice were injected intraperitoneally with D-Luciferin (150 mg/kg in PBS; Caliper Life Sciences, Hopkinton, MA, USA). After allowing 10 minutes for the distribution of D-Luciferin, mice were anesthetized (using 2% isoflurane), and bioluminescence was assessed using the IVIS imaging system 200 series (Xenogen Corporation, PerkinElmer, Waltham, MA, USA). The primary tumors were removed, imaged, fixed in formalin, paraffin-embedded, and sectioned (5 microns thick). Tumor cores were processed for histology and immunofluorescent staining of serial sections for various markers, using the antibodies detailed above. All fluorescent images of stained tumor sections were acquired using an Olympus DSU spinning disc confocal microscope and analyzed at the MD Anderson Flow Cytometry and Cellular Imaging Core Facility. For each antibody, staining was performed on tumors from at least three to four mice implanted with admixed tumor cells. Representative images are shown.

### Statistical analyses

Data are reported as mean ± SEM. *P*-values were calculated using Student’s unpaired two-tailed *t-test*, ^*^ *P* < 0.05, ^**^ *P* < 0.01, ^***^ *P* < 0.001, compared with the control. n.s., not significant (*P* ≥ 0.05).

## SUPPLEMENTARY MATERIALS



## Abbreviations

ANG-2: angiopoietin 2
CD: cluster of differentiation
CXCR-4: chemokine receptor type 4
DAPI: 4′:6-diamidino-2-phenylindole
DFX: desferrioxamine
DLL-4: delta like canonical Notch ligand 4
EGM-2: endothelial cell growth medium (supplemented with 2% fetal bovine serum and VEGF)
EMT: epithelial-mesenchymal transition
FLK1: fetal liver kinase 1
FOXC2: forkhead box C2
GFP: green fluorescent protein
H&E: hematoxylin and eosin
HEY-2: hairy/enhancer-of-split related with YRPW motif protein 2
HIF-1α: hypoxia-inducible factor 1 alpha
HMLE: human mammary epithelial cells immortalized with hTERT and SV40 large-T antigen
HMLER: RAS-transformed HMLE
hTERT: human telomerase reverse transcriptase
ITGB-3: integrin beta-3
KDR: kinase insert domain receptor
LDL: low density lipoprotein
LDLR: low density lipoprotein receptor
NOD/SCID: non-obese diabetic/severe combined immunodeficient mice
OPN: osteopontin
PDGF-β: platelet-derived growth factor-beta
RAS: rat sarcoma (viral oncogene homolog)
RFP: red fluorescent protein
SV40: simian virus 40
VEGF-A: vascular endothelial growth factor A
VEGFR-2: vascular endothelial growth factor receptor 2
