# Supplementary material for: Carcinoma cells that have undergone an epithelial-mesenchymal transition differentiate into endothelial cells and contribute to tumor growth
Source: Oncotarget. 2021 Apr 13;12(8):823–44. doi: 10.18632/oncotarget.27940 (PMC8057273; doi:10.18632/oncotarget.27940)
Supplement: Supplementary file 1 [file oncotarget-12-823-s001.pdf]

Carcinoma cells that have undergone an epithelial-mesenchymal transition differentiate into endothelial cells and contribute to tumor growth

SUPPLEMENTARY MATERIALS

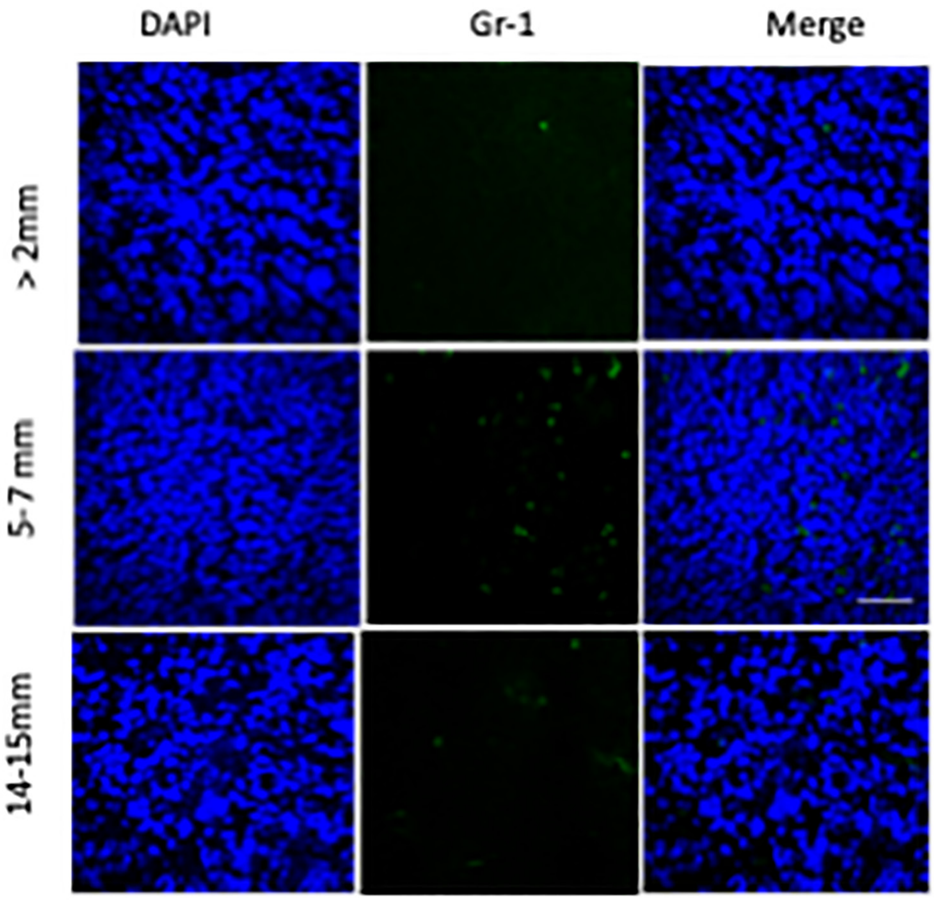

Supplementary Figure 1: Cells that have undergone EMT recruit Gr1+ MDSCs to tumors. The excised tumors (shown in Figure 1A) were fixed in formalin, paraffin-embedded, and sectioned prior to immunostaining with the indicated Gr-1 antibodies (green). Nuclei were counterstained with DAPI (blue). Scale bar, 100 µm.

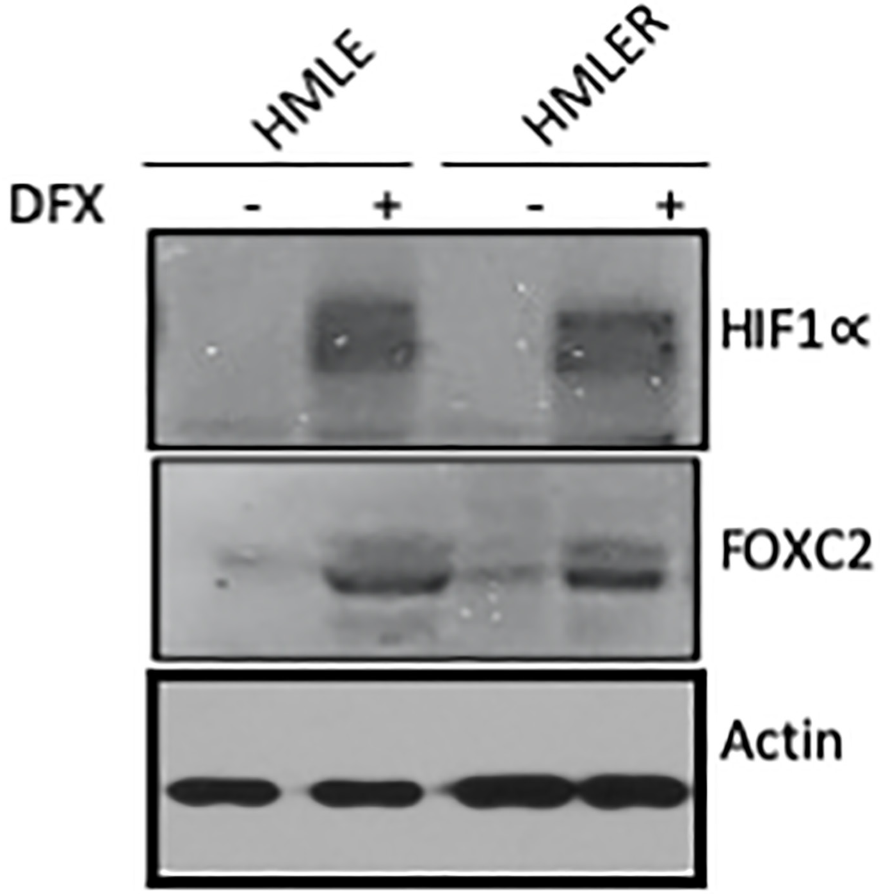

Supplementary Figure 2: HIF-1α and FOXC2 expression is elevated during hypoxia. Immortalized (HMLE) and RAS-transformed (HMLER) human mammary epithelial cells were plated in cell-specific medium. After 24 hours, the cells were treated either with vehicle or desferrioxamine (DFX), a hypoxia-mimetic, for 48 hours. Cells lysates were analyzed by immunoblotting with antibodies against HIF-1α and FOXC2.

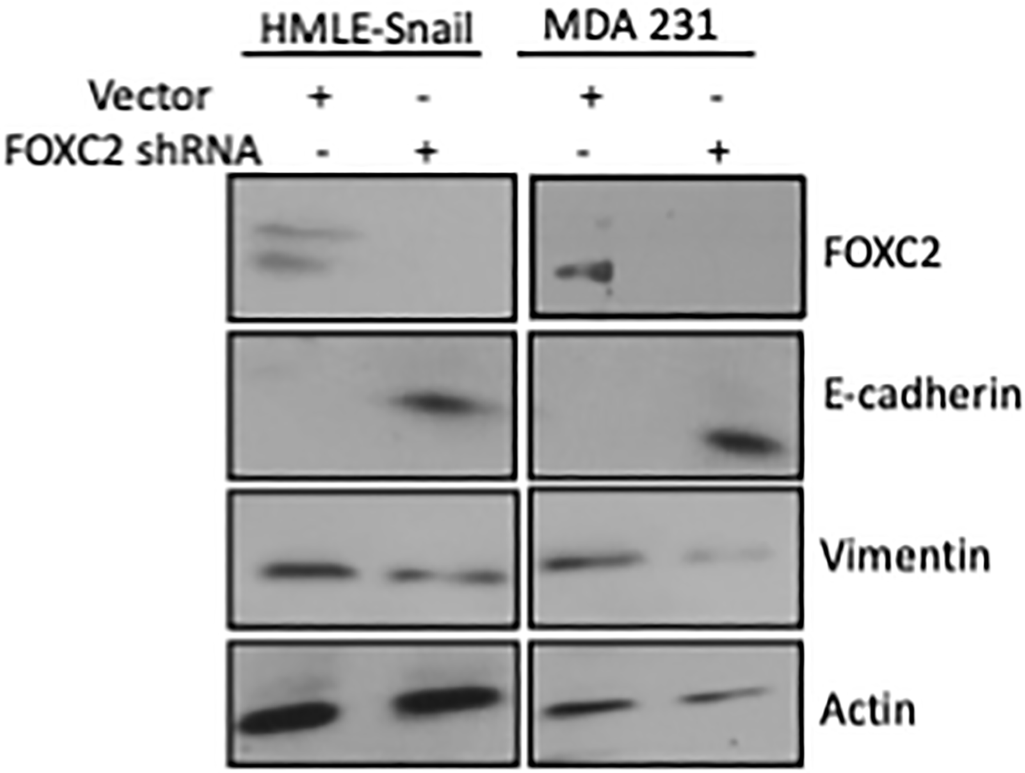

Supplementary Figure 3: FOXC2 plays an important role in EMT induction. Cell lysates from HMLE-Snail-shControl, HMLE-Snail-shFOXC2, MDA-MB-231-shControl, and MDA-MB-231-shFOXC2 cells were analyzed by immunoblotting with antibodies against EMT markers such as FOXC2, E-cadherin, and vimentin. β-actin was used as a loading control.
